# Sound and Soundscape in Restorative Natural Environments: A Narrative Literature Review

**DOI:** 10.3389/fpsyg.2021.570563

**Published:** 2021-04-26

**Authors:** Eleanor Ratcliffe

**Affiliations:** Faculty of Health and Medical Sciences, School of Psychology, University of Surrey, Guildford, United Kingdom

**Keywords:** soundscape, nature sounds, restorative environments, attention restoration, stress recovery

## Abstract

Acoustic experiences of nature represent a growing area in restorative environments research and are explored in this narrative literature review. First, the work surveyed indicates that nature is broadly characterized by the sounds of birdsong, wind, and water, and these sounds can enhance positive perceptions of natural environments presented through visual means. Second, isolated from other sensory modalities these sounds are often, although not always, positively affectively appraised and perceived as restorative. Third, after stress and/or fatigue nature sounds and soundscapes can lead to subjectively and objectively improved mood and cognitive performance, as well as reductions in arousal, although some inconsistencies in findings are observed. Fourth, theoretical frameworks of restorative environments would benefit from inclusion of acoustic environmental properties such as sound intensity or frequency. Fifth, findings regarding positive, learned semantic associations with nature have arisen as a result of recent work on sounds and restoration. This represents another important area of potential theoretical development for broader restorative environments research.

## Introduction

There is an abundance of literature regarding the ability of certain settings, termed “restorative environments,” to facilitate recovery from everyday cognitive fatigue, negative mood, and stress (Collado et al., 2017). Much attention has been paid to the restorative value of natural environments in particular (Hartig et al., 2014). Studies on these topics tend to focus on visuo-spatial experience of environments, utilizing stimuli such as photographs, videos, and slideshows, but environments are not experienced through vision alone. There is growing interest in and call for study of non-visual aspects of restorative environments, including sound, smell, and touch (Conniff and Craig, 2016; Iyendo, 2016; Franco et al., 2017; Aletta and Kang, 2019; Sona et al., 2019; Schebella et al., 2020). Such work is important to ensure that the research field remains relevant to individuals with visual impairment (Shaw et al., 2015; Bell, 2019a,b) and to maximize extended reality presentations of environments, e.g., through virtual or augmented reality (Depledge et al., 2011).

While research on touch and smell in restoration remains limited, nature sounds and natural soundscapes are increasingly identified as important ecosystem services that can aid psychological restoration as well as well-being more broadly (Francis et al., 2017). Here soundscape is defined as the acoustic environment as perceived, understood, and/or experienced by people, in context (see International Organization for Standardization, 2014). However, the theories that seek to explain why certain environments facilitate restoration focus primarily on visual experience (see Ulrich, 1983; Kaplan and Kaplan, 1989). The first step in better integrating sound and soundscape into our theoretical understanding is to examine and review the available literature.

**Figure 1:**
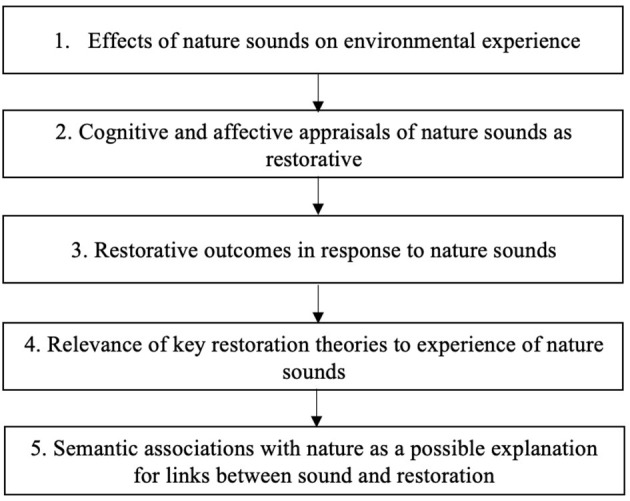
Flowchart summarizing the key topics of interest in this literature review.

A systematic review by Aletta et al. (2018) has identified links between positive urban soundscapes (which may also include nature sounds) and health and well-being, including stress recovery. Given the emphasis on nature within restorative environments (see Hartig et al., 2014), the present narrative literature review focuses on evidence for positive psychological experiences of nature sounds and soundscapes specifically, and in particular how listening to these can generate perceptions and outcomes of restoration from stress and fatigue. This review has five key objectives, summarized in Figure 1. First, it explores literature regarding the impact of nature sounds on perceptions and experiences of wider natural environments. Second, it examines evidence regarding cognitive and affective appraisals of nature sounds and their contributions to overall perceptions of restorative environments. Third, literature regarding restorative outcomes in response to nature sounds is assessed. Fourth, the relevance of key restoration theories to this topic is examined and areas where these theories are limited are identified. Fifth, a possible new theoretical area of interest—semantic associations with nature—is discussed and exemplified by recent acoustics research.

## Sounds Are Important for Environmental Experiences of Nature

Peace and quiet are important aspects of being in nature but this does not mean the presence of complete silence—rather, it can relate to the concept of relative tranquility, or reduction in sounds from the built environment and the opportunity to hear pleasant sounds of nature (De Coensel and Botteldooren, 2006; Pheasant et al., 2008).

Qualitative studies describe exposure to natural environments as a positively regarded, multi-sensory experience, whereas a lack of such multi-sensory aspects is regarded negatively. For example, following qualitative interviews with 20 wildlife tourists, Curtin (2009, p. 461) reported that participants experienced a heightened sensory awareness after wilderness trips to locations in Spain and USA: “I have seen and heard things in the natural world that I didn't know even existed. It was as if my senses were coming alive…” Curtin describes the sensory dominance of vision in the wildlife tourism experience but notes that it is experienced in the context of other sensory modalities such as sound and smell. In their qualitative study, Fredrickson and Anderson (1997, p. 31) found that a sample of 12 women reported direct experience of the sounds of nature as a particularly meaningful aspect of wilderness trips to Minnesota and Arizona, USA. As one participant observed, “It was so incredible being able to hear the birds, yeah, and just the crunching of animals all around us… The sounds of the forest, the snapping of the twigs, hearing the tiny sigh of the wind through the treetops at night.”

In a study of participants awaiting treatment at a stress clinic in Sweden, Kjellgren and Buhrkall (2010, p. 470) qualitatively explored differences in restoration after direct exposure to Swedish woodland and exposure to the same environment mediated through photographs. In the mediated exposure condition themes regarding an absence of sensory input were prevalent; e.g., “Missing the smells and sounds.” The absence of auditory input was related to potentially negative affective states such as loneliness (“I feel a lonely quietness”) although another participant framed the lack of sound in a more positive way: “Peace and quiet.” In contrast, themes from the direct exposure condition reflected increased sensory awareness (“After awhile I hear more and more sounds of nature”… “My senses feel heightened now;” Kjellgren and Buhrkall, 2010, p. 469). These data suggest that experiencing the mediated natural environment, lacking in sound, was unsatisfactory for some participants to the extent that it caused varying perceptions of stress, boredom, and lack of concentration. Kjellgren and Buhrkall (2010) suggest that this may be due to a lack of presence in the mediated environment.

This perspective is supported by qualitative participant comments in an otherwise quantitative study conducted by Annerstedt et al. (2013) regarding virtual reality experience of a forest with and without nature sounds. When the sounds were absent the forest was regarded as unsettling, as though something was missing. In quantitative analysis of data provided by Swedish residents, Grahn and Stigsdotter (2003, p. 7) observed that areas of green space such as quiet parks, rated as helpful when feeling stressed or worried, do not lack sounds completely but feature “sounds of the wind, birds, water, etc.” Similarly, Björk et al. (2008, p. 3) note that serenity and lushness are desirable characteristics of natural environments, where serenity is defined as “sounds of wind, water, birds, and insects” and lushness as “a place rich in species.”

The sounds of nature are an integral part of environmental experience and appreciation (Mace et al., 2004) and quantitative studies also show that they play an important role in the way natural environments are perceived. For example, supplying nature sounds alongside visuospatial nature stimuli can significantly enhance positive appraisals of the setting, including preference and perceived restorativeness (e.g., Anderson et al., 1983; Jahncke et al., 2015; Franěk et al., 2018; Zhao et al., 2018; Zhu et al., 2020]. This may be due to an increased sense of presence in the environment generated by greater sensory input and awareness as a result of the presence of sound. Support for this argument comes from a body of qualitative work, described below, in which the experience of natural sounds is expressed as a desirable and immersive aspect of being in nature.

Overall, exposure to aspects of nature beyond the purely visual—including sounds—appears related to a greater sensory awareness, immersion in, and sense of presence within nature. This immersion is described in positive terms by participants in qualitative studies such as Curtin (2009) and Kjellgren and Buhrkall (2010), whereas the lack of immersion offered by visual experience of nature only is seen as less positive in comparison (Annerstedt et al., 2013). These findings suggest that natural sounds may offer benefits to restorative perceptions and experiences by affording a greater sense of realism and immersion in nature.

## Appraisals of Nature Sounds as Pleasant, Relaxing, aAnd Potentially Restorative

Perhaps the largest body of literature on human experiences of natural sounds relates to how they are affectively and cognitively appraised. This literature is both qualitative and quantitative, and these two bodies of work are discussed separately here.

### Qualitative Approaches to Appraisals of Natural Sounds

Qualitative research indicates a relationship between the presence of natural sounds and a state of positive affect. In semi-structured interviews with rural-dwelling Portuguese participants, Pereira et al. (2005, p. 26) revealed a theme of “the feeling of joy provided by bird songs.” Similarly, Modelmog (2002) interviewed farmers' wives in Ammerland, Germany, about their relationships with nature. A participant associated listening to birdsong with a positive affective state: “In my garden there blooms a sunflower. […] Sometimes a bird sits on it and sings. This is happiness to me (Modelmog, 2002, p. 120).” Curtin (2009) also reported that participants associated wildlife sounds with changes in psychological states. For one participant, birdsong was associated with a shift from negative to positive affect: “When you have not been sleeping and you wake up very early and you hear the dawn chorus and you hear the birds, you can suddenly in seconds feel uplifted…” (Curtin, 2009, p. 469).

In a series of semi-structured interviews, Ratcliffe et al. (2013) found that members of the British public generally associated the sounds of nature (e.g., water, wind, and birdsong) with perceived restorative experiences such as pleasure, relaxation, and escape from everyday concerns. Kjellgren and Buhrkall (2010, p. 469) reported that participants in their study responded to the sounds of nature with positive affective appraisals and perceptions of reduced arousal, with one participant noting, “The singing of the birds makes me feel relaxed” and another describing “calming sounds” heard in nature. Similarly, in Cerwén's et al. (2016) qualitative study, Swedish patients recovering from stress perceived nature sounds in a rehabilitation garden as a source of pleasure, relaxation, and restoration. While much research on restoration focuses on green space, Nicolosi et al. (2020) identify coastal soundscapes as positive predictors of perceived restoration.

The restorative experiences of blind and visually impaired individuals in nature has been largely neglected in environmental restoration literature, perhaps due to the strong visuo-spatial focus of existing studies, but this body of work can tell us a great deal about perceptions of natural sounds. Shaw et al. (2015) specifically examined the experience of visually impaired individuals in nature via semi-structured interviews. Thematic analysis revealed perceived restoration arising from sounds as a key theme of experiences in nature. One participant, Helen, noted that, “...you hear a lot of birds. That, that gives you a tremendous feeling of well-being […] a much more peaceful feeling than you have when you are at home” (Shaw et al., 2015, p. 8). In the context of her wider project “Sensing Nature,” Bell (2019a,b) also reported that individuals living with sight impairment used sound as means of connecting with nature, and particularly with wildlife, and experienced positive affective states such as pleasure, freedom, and reduced vulnerability as a result.

### Quantitative Approaches to Appraisals of Natural Sounds

The work referenced above indicates that natural sounds are often related to affective states of pleasure and relaxation. Quantitative evidence suggests a similar story and contrasts these positive appraisals with more negative evaluations of anthropogenic sounds. For example, Kariel (1980) recruited individuals from the general public and a mountaineering population and found that both samples considered nature-based sounds of wind, water, wild native fauna (including birds and insects) pleasing or agreeable, whereas the sounds of people and technology were considered neutral or acceptable at best and annoying at worst. Both samples rated the top three sounds (wind, water, and wild animals) equally pleasant. Similarly, Anderson et al. (1983) observed that sounds such as wind, insects, and birdsong were most preferred amongst a range of natural, human, and mechanical sounds.

Assessments of these sounds as pleasant has implications for how beneficial they may be to listeners. Medvedev et al. (2015) integrated subjective ratings of environmental sounds and objective measures of stress recovery to show that ratings of natural sounds as pleasant were related to their ability to aid recovery from stress. In a questionnaire study of Swedish residents, Hedblom et al. (2017) found that women and older participants in particular reported finding nature sounds (such as birdsong and wind in leaves) calming, suggesting potential interactions between sound appraisal and demographics or individual differences.

Using the purpose-developed Perceived Restorativeness Soundscape Scale (PRSS), Payne (2013) differentiated between the perceived restorativeness of urban, urban park, and rural soundscapes in a lab setting, with the rural soundscape (comprising birds, water, and wind) scoring most highly. Similarly, Emfield and Neider (2014) observed that natural sounds of the sea and seagulls were rated as more relaxing than sounds from the urban environment. Even when differences in positive appraisals were controlled for, Kryzwicka and Byrka (2017) found that nature soundscapes were perceived as more restorative. These findings indicate that typical sounds and soundscapes of nature are considered more restorative than those from the built environment, echoing the distinction found between visuo-spatial natural and urban environments (Hartig et al., 2014).

### Not All Sounds in Nature Are Perceived as Pleasant

There is, however, evidence to suggest that not all nature sounds are regarded equally positively. In a ratings study of fifteen natural sounds, Björk (1985) found that the songs of chaffinches and other songbirds were rated as more pleasant than the calls of lapwings or gulls. Bradley and Lang (2007) measured 167 sounds on scales of pleasure, arousal, and dominance, of which 21 sounds were from natural sources such as animals (including birds), water, and wind. Some natural sounds, such as water and birds, scored relatively high on pleasure while others, such as growling, were rated as less pleasant, indicating that although natural sounds may generally be perceived as pleasant there is variation depending on the type of sound and its source. Similar findings are reported by Hume and Ahtamad (2013), in which wave sounds and birdsong were rated as very pleasant but the sound of foxes was not. Work by Ratcliffe et al. (2013, 2016, 2020) shows that there is variation even within a single category of nature sound (bird songs and calls): songbirds are qualitatively and quantitatively regarded as more pleasant, relaxing, and potentially restorative than birds which make rough, noisy, and simple calls, or those which have negative meanings or associations. Zhao et al. (2020) have linked crow sounds specifically to lower evaluations of the perceived restorativeness of park soundscapes, while woodpeckers and sparrows are related to more positive evaluations. These findings suggest that variations in preference and perceived restorative value exist even between types of nature sound within the same category. Moreover, combinations of natural sound that reflect biodiversity are also positively regarded. Hedblom et al. (2014) observed that combinations of bird sounds were rated as more pleasant than the sounds of a single species, which may be linked to positive perceptions of biodiversity. This is supported by findings that locations judged to be rich in bird sound are also perceived as more restorative (Fisher et al., 2021), including when such sounds are experimentally manipulated (Ferraro et al., 2020).

## Nature Sounds Can Lead to Restorative Outcomes

In many experimental studies that examine restorative outcomes, sounds have been included as part of the experimental stimuli (audible *in situ* or through audio-visual recordings) but their contributions to the restorative experience were not specifically examined (e.g., Ulrich et al., 1991; Hartig et al., 2003; van den Berg et al., 2003; Berman et al., 2008). A growing body of literature has set out to address this. In the following section this is reviewed in two parts: research relating to subjectively measured restoration, and that relating to objective measures (i.e., change in physiological state and/or performance on cognitive tasks).

### Subjectively Measured Restoration

Jahncke et al. (2011) examined the restorative effect of a 7-min exposure to audio-visual media of a river, audio media of a river only, silence, or high office noise. Participants who experienced audio-visual media of the river self-reported having more energy than those who experienced only river only or high noise conditions. Both audio-visual and audio exposure to the river media resulted in higher self-reported motivation to work than exposure to office noise. This suggests that experience of nature sounds contextualized by visuals may produce self-reported restorative outcomes, although Ma and Shu (2018) have reported restorative effects of nature sounds independently of visual stimuli.

Studies exploring the restorative effects of natural sounds, separate from visual experience, have until recently been relatively limited. Goel and Etwaroo (2006) observed that exposure to a recording of birdsong combined with classical music significantly reduced self-reported depression and anger in a sample of University students, both depressed and non-depressed. While the findings suggest that listening to birdsong, among other sounds, can have rapid effects on self-reported mood, the study does not dissociate the effects of birdsong from the effects of music, a stimulus which is well-known to induce affective change (see McDermott, 2012, for a review). In a laboratory experiment, Benfield et al. (2014) exposed participants to a stress- and negative affect-inducing video and then to one of four conditions: natural sounds (birdsong and rustling leaves); natural sounds plus traffic; natural sounds plus voices; or a control condition with no audio present. Only in the natural sounds condition did participants show improvements in mood, while participants in the other three conditions showed either declines or non-significant increases. Effects on arousal were not investigated in this study; however, Ma and Shu (2018) examined responses to nature sounds within a simulated open-plan office and found that water and birdsong sounds significantly aided recovery from self-reported annoyance as well as fatigue.

### Objectively Measured Restoration

In line with physiological effects of soundscapes more broadly (see Erfanian et al., 2019, for a review), studies that objectively measure the effects of nature sounds on stress recovery reveal mixed results. On the one hand, Annerstedt et al. (2013) observed that experiencing a virtual reality forest environment with birds and water sounds aided recovery from a social stress task (measured via change in heart rate variability) to a greater extent than experiencing the forest environment without sounds or no environmental experience. Participants who listened to nature sounds for 7 min in a waiting room setting showed significantly reduced pulse rate and muscle tension, whereas those who listened to classical music or silence did not (Largo-Wight et al., 2016). Alvarsson et al. (2010) found that stress recovery, as measured by change in skin conductance level (SCL), was significantly greater when participants listened to birdsong and water sounds mixed together vs. loud traffic noise. Recovery from stress tended to be faster, although not significantly greater, in the nature condition than in response to quiet traffic noise or ambient environmental noise. Hedblom et al. (2019) reported no significant differences in stress recovery between three sound conditions (birdsong, traffic, and birdsong + traffic).

Alvarsson et al. (2010) reported faster recovery in the nature condition than the low noise condition even though these were presented at the same sound pressure level (50dB LAeq, 4 min), suggesting that differences in the loudness vs. quietness of an acoustic environment may not be completely responsible for stress recovery. Instead, they suggest that the perceived pleasantness of the sounds may also be relevant and could pertain to their semantic content rather than merely their acoustic properties. In an extension of this work where sound pressure levels were controlled at an average of 64 dB SPL^1^ across conditions, Medvedev et al. (2015) observed faster decreases in skin conductance level following stress when participants were exposed to bird and water sounds, vs. sounds from the built environment.

Similar results are reported in studies of objective psychophysiological responses to natural sounds, even in the absence of a prior stress/fatigue condition. For example, Gould van Praag et al. (2017) found that participants who listened to familiar nature sounds showed better attentional monitoring and increased parasympathetic nervous activity than those who listened to artificial sounds. Jo et al. (2019) found that participants who experienced sounds of the forest displayed reduced signs of physiological arousal (i.e., reduced sympathetic nervous system activity) as compared to those who experienced urban sounds. Li and Kang (2019) found that listening to 5-min nature sound recordings (birdsong, ocean waves) led to reductions in certain signs of physiological arousal, including heart rate and respiration frequency and depth, whereas street and traffic soundscapes did not. Contrastingly, Hume and Ahtamad (2013) observed small but significant reductions in heart rate after listening to short (8-s) clips of unpleasant sounds.

Literature regarding effects of natural sounds on objective measures of cognitive performance, or cognitive restoration after fatigue, is also somewhat contradictory. On one hand, Emfield and Neider (2014) reported no significant differences in change in cognitive performance (as measured via pre- and post-exposure administration of a battery of cognitive tasks) as a result of listening to ocean and bird sounds, vs. urban sounds. Abbott et al. (2016) reported only marginally significant restorative effects of nature sounds on cognitive performance as measured via a backwards digit span task (BDST). On the other hand, Van Hedger et al. (2019a) reported significant improvements in cognitive performance (as measured by a composite dual n-back task and BDST) among participants exposed to nature sounds, as opposed to urban sounds, although surprisingly no such effects were found on change in affect. Among samples of school children, Shu and Ma (2019) found that listening to nature sounds (birdsong, water sounds) led to faster responses on a sustained attention to response task (SART) and increased performance on a digit span task (DST). In an *in situ* study in China where sound recordings were experimentally manipulated, Zhang et al. (2017) found that participants exposed to nature-based sounds showed greater attention restoration (as measured via performance on a mental arithmetic task) than those exposed to traffic or machinery sounds.

The studies reviewed above indicate that natural sounds can, in some cases, generate restorative outcomes in terms of affect, psychophysiological arousal, and cognition. These can occur separately from visual exposure to nature but may be enhanced by the presence of visual stimuli.

## Theoretical Approaches to Sounds and Restoration

Since the 1980s two key theories have attempted to explain why certain environments, and particularly nature, can facilitate restoration. These are attention restoration theory (ART; Kaplan and Kaplan, 1989; Kaplan, 1995) and stress reduction theory (SRT; Ulrich, 1983; Ulrich et al., 1991). As mentioned at the start of this review these theories focus predominantly on visual experience of natural environments. Ulrich (1983, p. 86) observes that “many sounds and smells in natural settings surely also influence our feelings,” but ultimately focuses on visuo-spatial and aesthetic properties of the environment that can influence affective appraisals and reductions in arousal. These include visual complexity, pattern, depth of scene, surface texture, deflected vistas, and affordances of resources (e.g., water) and threats (e.g., predatory animals). A more recent processing fluency account (PFA; Joye and van den Berg, 2011) challenges some of the psycho-evolutionary principles behind SRT but this too is framed in terms of ease of processing of visual environmental properties.

ART (Kaplan and Kaplan, 1989; Kaplan, 1995) proposes that restoration is driven by cognitive experiences of soft fascination or effortless attention to the environment, a sense of psychological escape or being away, spatial extent, and person-environment compatibility. While these concepts should, in principle, apply to different types of sensory experience, in practice the theory relies heavily on visual examples of such experiences (e.g., experiencing fascination by looking at natural phenomena) to illustrate relevant concepts. Sounds are not mentioned in the original theoretical work, yet visually complex scenes can be represented through acoustically complex soundscapes (Andringa and Lanser, 2013) and in developing a measure of perceived restorativeness of soundscapes Payne (2013) has shown that ART can be applied to acoustic experiences. Even the acoustic and aesthetic properties of individual sounds (bird songs and calls) are related to assessments of perceived restorativeness (Ratcliffe et al., 2020). Work by Qiu et al. (2021) on natural soundscapes during the COVID-19 pandemic suggests that acoustic features of an environment can impact directly on appraisals of ART constructs of extent and fascination, while being away and compatibility may be indirect products of these appraisals. It seems timely to evaluate and update key theories of restoration in order to include acoustic properties in the same way that low-level visual features of environments are considered (see, e.g., Schertz and Berman, 2019).

## Associations With Nature: a New Avenue for Theoretical Development?

A concept that is alluded to in SRT (Ulrich, 1983) but is otherwise not greatly explored is the potential relationship between semantic properties specific to an individual and restorative perceptions and outcomes in response to nature. Such properties might include associations, memories, or meanings otherwise linked to the environment (Stigsdotter et al., 2017). Researchers studying the restorative effects of natural sounds and soundscapes have argued for a more interpretative, constructionist approach to how individuals perceive and respond to these environments; i.e., that individuals experience natural soundscapes as a result of both bottom-up, perceptually driven processes and those that are top-down, based on existing preferences, attitudes, and cognitions (Payne, 2008; Ratcliffe et al., 2016). Compatibility between individuals and their soundscapes is also emphasized as a key predictor of perceived restoration by Qiu et al. (2021).

On listening to nature sounds individuals may visualize their own imagined natural environments (Ratcliffe et al., 2016; Bates et al., 2020). There is experimental evidence regarding influences of imagination and learned association on perceptions of the restorativeness of sounds. Listening to pink and white noise reduced self-reported feelings of exhaustion when participants were told that it was the sound of a waterfall, as opposed to a machine, despite the sound itself remaining objectively the same (Haga et al., 2016). Similarly, Van Hedger et al. (2019b) found that preference for natural over urban sounds was dependent on the sounds being recognizable as from these respective categories, and that when this was not recognizable the acoustic properties that characterized these sounds were not in themselves predictors of preference. This recent body of work emphasizes the contribution that semantic, associative interpretations of nature make to restorative perceptions and outcomes, over and above any such effects resulting from perceptual, sensory experiences. As a next step, restoration researchers could incorporate this constructivist approach into theoretical models. This may be achieved by focusing on individual differences in environmental identities, implicit associations with environments or environmental stimuli, perceived rather than objective sources of environmental stimuli, and aspects of individual bonds with place.

## Discussion and Conclusions

A growing body of work demonstrates restorative perceptions of and outcomes associated with listening to nature sounds, in line with wider evidence that visuo-spatial experiences of nature can benefit psychological well-being (Hartig et al., 2014). As outlined in this review, birdsong, wind, and water are often considered characteristic of pleasant, tranquil natural environments. The presence of these sounds can enhance immersion and sense of presence in visual or other virtually mediated environments and increase positive appraisals of these settings. These sounds are typically perceived as pleasant and calming although variation in such appraisals exists between different *types* of natural sounds. While evidence for restorative *perceptions* of nature sounds is broadly consistent, the evidence for restorative *outcomes* (both cognitive and affective) arising from such exposure is somewhat inconsistent. This may be a result of the different methodologies used in these studies, which are themselves still limited in number in comparison to research on visual experience of nature.

This review has also considered the attention given to acoustic stimuli by key restorative environments theories; i.e., ART (Kaplan and Kaplan, 1989) and SRT (Ulrich, 1983). It is notable that vision and visual examples from nature dominate the original works in which these theories were set out. It is evidently the case that natural sounds can lead to some form of restoration, be it subjectively perceived or objectively measured, and therefore the key theories will need to change to better accommodate acoustic environmental factors in the same way that they do visual properties of environments. For example, in addition to including surface texture and depth of scene as SRT does, a modified theory or model might also include sound intensity and frequency. Finally, work by Haga et al. (2016) and Van Hedger et al. (2019a) suggests that the semantic value of natural sounds (i.e., as natural, and therefore positive) may inform restorative perceptions, beyond mere evaluation of perceptual properties. Greater focus within restoration theory on the meanings that people associate with environments is overdue and may serve to better explain how and why different settings—within and beyond nature and experienced through a variety of sensory means—can support psychological well-being.

## Author Contributions

ER conducted all aspects of this literature review.

## Conflict of Interest

The author declares that the research was conducted in the absence of any commercial or financial relationships that could be construed as a potential conflict of interest.

## Footnotes

^1^Further information on the acoustic presentation, e.g., A-weighted SPL, is not given in the cited paper and therefore cannot be commented upon here.
